# Optimization of solar water pumping systems through a combined approach based on MPPT-Bat and DTC

**DOI:** 10.1371/journal.pone.0309330

**Published:** 2024-12-30

**Authors:** Abdelilah Hilali, Mouncef El marghichi, Mohamed Makhad, Azeddine Loulijat, Najib el Ouanjli, Mahmoud A. Mossa, Mishari Metab Almalki, Thamer A. H. Alghamdi

**Affiliations:** 1 Faculty of Sciences, Moulay Ismail University, Meknes, Morocco; 2 Intelligent Systems Design Laboratory(ISDL), Faculty of Science, Abdelmalek Essaadi University, Tetouan, Morocco; 3 Department of Electrical Engineering, ENSAM Rabat. Mohammed V university, Rabat, Morocco; 4 Laboratory of Mechanical, Computer, Electronics and Telecommunications, Faculty of Sciences and Technology, Hassan First University, Settat, Morocco; 5 Faculty of Sciences and Technology, Hassan first University, Settat, Morocco; 6 Electrical Engineering Department, Faculty of Engineering, Minia University, Minia, Egypt; 7 Department of Electrical Engineering, Faculty of Engineering, Al-Baha University, Alaqiq, Saudi Arabia; 8 Electrical Engineering Department, Faculty of Engineering, Al-Baha University, Al-Baha, Saudi Arabia; 9 Wolfson Centre for Magnetics, School of Engineering, Cardiff University, Cardiff, United Kingdom; Vellore Institute of Technology, INDIA

## Abstract

This paper investigates enhancing the efficiency of solar water pumping systems (SWPS) by implementing a Maximum Power Point Tracking technique based on the Bat Metaheuristic Optimizer (MPPT-bat) for the photovoltaic generator (PVG) side, coupled with Direct Torque Control (DTC) for the induction motor powering the pump. Unlike traditional techniques, which make no compromise between tracking speed, oscillation and robustness. The integration of the MPPT-bat represents a significant advance, making it possible to improve PVG performance whatever the weather conditions. The main objective remains to improve the energy efficiency of this type of application by maximizing the electrical power allocated to the SWPS. At the same time, a DTC controller applied to the pump motor aims to optimize the use of the energy generated by the MPPT-bat. This intelligent approach adjusts the motor power according to the power extracted from the PVG, thus avoiding inappropriate profiles for the pumping system. The study confirms that optimizing SWPS using this approach based on MPPT-bat and DTC, significantly improves overall performances in terms of tracking error, oscillations, tracking speed and robustness, promotes more efficient pump rotation and, consequently, increases the flow rate of pumped water, and that these improvements persist under different climate conditions.

## 1. Introduction

Faced with environmental and energy challenges, sustainable agriculture encounters a major problem in regions where rainfall is insufficient to meet water requirements. In this context, the implementation of pumping systems becomes imperative to ensure an adequate water supply [1, 2]. Historically, solutions to this water shortage have often relied on the use of diesel or butane pumps. However, the advent of solar-powered pumping systems today represents both an environmentally friendly and economically advantageous alternative, marking a significant departure in energy choices for agricultural irrigation as reported in [3–5]. The viability studies carried out unequivocally confirm the numerous advantages of solar water pumping systems over their conventional counterparts in [6, 7]. This finding establishing their superiority makes solar systems an undisputed priority for meeting the growing water needs of agriculture, particularly in areas characterized by limited water resources. By embracing this transition to sustainable energy solutions, agricultural areas can not only overcome the challenges of limited water availability, but also make a significant contribution to preserving the environment by reducing their carbon footprint and promoting a more responsible use of natural resources [8]. The widespread adoption of solar-powered pumping systems is therefore a strategic and beneficial move, both environmentally and economically for the future of agriculture.

The challenges facing the world’s energy supply are extremely diverse, ranging from continued dependence on fossil fuels to major environmental concerns linked to greenhouse gas emissions. The urgent need to reduce these emissions while ensuring a stable energy supply has highlighted the crucial importance of renewable energies [9, 10]. Among these alternative sources, solar energy stands out for its inestimable potential, particularly in regions of the globe with abundant sunshine. The judicious exploitation of this solar resource offers a significant opportunity to help mitigate the effects of climate change, while meeting growing energy needs in a sustainable way Indeed, the increased use of solar energy represents a promising way of overcoming current and future energy challenges, marking a transition towards more sustainable, environmentally friendly energy practices [11]. The increasing use of solar energy as a viable and renewable energy solution is becoming an essential strategy for tackling the complex energy challenges facing the world. This transition to more extensive use of solar energy will not only help to reduce greenhouse gas emissions, but will also help to create a more resilient, promising and sustainable energy future on a global scale [12].

Solar energy is emerging as an innovative source with multiple applications, revolutionizing diverse sectors such as electrification of remote regions [13], powering structures [14], as well as transport and agriculture [15]. Among these applications, solar water pumping systems are particularly significant, offering the possibility of irrigating agricultural fields far from traditional power grids, thus making a substantial contribution to promoting sustainable agriculture and guaranteeing local food security. Exploring these diverse applications in detail, it becomes apparent that solar energy stands out for its exceptional versatility. However, to fully optimize the use of solar energy, specific technical challenges need to be overcome. These include the continuous improvement of technologies such as solar cells and solar trackers, with particular emphasis on maximum power point tracking (MPPT) [16], the control of pumping machines [17, 18], and, DC-DC converters [19, 20]. The latter devices play a crucial role in real-time adjustment of the static DC-DC converter duty cycle to manage the amount of energy available in the PV grid, thus maximizing solar energy capture [21].

It is imperative to highlight that advances in *MPPT* technology in these areas are fundamental to significantly increasing the efficiency of solar energy, and to solving practical problems that arise when it is used on a large scale. Ongoing research and innovative development in solar technologies are crucial to unlocking the full potential of solar energy and driving a global energy transition to renewable sources. Traditional MPPT methods, such as P&O [22] and INC [23], are widely used to optimize GPV power generation. They adjust electrical parameters, such as voltage or current, to track the MPP. The tracking speed of conventional MPPTs can be slow, resulting in insufficient responsiveness to changes in weather conditions, such as temperature or irradiance [24], and therefore sub-optimization of energy. In fact, these traditional techniques always have a trade-off between tracking speed and oscillations, and these two performances are inverse. In fact, increasing the tracking speed leads to significant oscillations around MPP, which is not only inefficient, but can also lead to premature degradation of system components. To overcome these problems, traditional variable-step MPPT techniques are used in [25, 26]. However, another major drawback is that these techniques do not always distinguish between the local power point and the global power point in the case of partial shading, which limits their ability to maximize the energy produced by photovoltaic panels under different conditions [27]. To overcome these limitations, the use of artificial intelligence-based techniques, such as neural networks [28], and genetic algorithms [29], is proving effective. However, learning and testing phases are essential to adapt these techniques to the specificities of solar panels, and powerful computers are required to solve tracking speed problems.

The transition to clean energy sources is imperative. It requires MPPT optimization techniques that are at the cutting edge of current research. These techniques are based on the search for optimal solutions using natural phenomena such as Particle Swarm Optimization (PSO) [30], Frog-Leaping [31], Firefly Based Ant Colony (FBAC) [32] and MPPT-bats. These techniques require no training other than prior testing on PVGs, and are adaptable to all kinds of solar applications. This research paper discusses the bat metaheuristic method, which offers an innovative approach, drawing on biological concepts to solve the problems described above. This approach represents a significant advance in optimizing the energy efficiency of solar water pumping systems. By adjusting electrical parameters as a function of distance from the point of maximum power, this method guarantees rapid responsiveness to changing weather conditions, while minimizing unwanted oscillations. In addition, it makes a more precise distinction between local and global power points, enabling more accurate and efficient adaptation to actual conditions [33]. Applying the bat method to a solar water pumping system enables more optimal use of solar energy, thus contributing to more sustainable management of energy resources.

Solar pump system optimization depends heavily on control of the induction machine, which contributes significantly to energy efficiency. This control covers all components, from the photovoltaic generator to the motor pump. The latter assumes particular importance in meeting crop water requirements via the hydraulic system. An inappropriate pumping profile can lead to significant pressure losses and problems that can result in damage to the system [34]. To ensure the safety of these installations, a pump motor control strategy is required. Here’s where direct torque control (DTC) is particularly relevant. The DTC controller has the advantage of being relatively simple to implement, requiring fewer hardware components and sensors than other control techniques [35]. This simplicity facilitates maintenance and increases the system’s durability. As a control system, DTC provides fast, precise control of solar pump motors. Its distinctive feature derives from its ability to adjust motor torque in real time as conditions require, without the need for a complex external control loop. Integrating the DTC into solar water pumping systems offers the possibility of maximizing energy efficiency while ensuring a reliable supply of water for crop irrigation.

The aim of this research paper is to improve the energy efficiency of solar water pumping systems by using a two-stage process. In the first phase, attention is focused on the maximum extraction of energy generated by PVGs using the MPPT-Bat technique. This method adjusts the PVG parameters in real time to extract the fully of available energy. The second part of the study focuses on the application of DTC control to ensure optimum exploitation of the energy generated by the PVGs through the induction machine. As such, the DTC controller is deployed to ensure optimum performance of solar water pumping systems by precisely and responsively controlling motor torque, helping to ensure efficient use of available solar energy. This integrated approach, combining Bat optimization and DTC control of pumping motors, aims to maximize the energy efficiency of these systems crucial to water supply in agricultural environments.

This paper makes several notable contributions, which are highlighted as follows:

- An approach to optimize SWPS using MPPT-Bat approach on the PVG side and DTC on the induction motor side.
- Under different weather conditions, the MPPT-bat technique is characterized by the ability to extract maximum power from PVG side. Meanwhile, the DTC optimize the use of the energy generated by the PVG. It provides precise, responsive control of the pump motor.
- Following a description of the system, a model of the entire SWPS is presented.
- A carefully selected setpoint profile, derived from the evolution of the actual irradiance recorded by an experimental weather station, is used to evaluate the performance of the two proposed techniques.
- The results highlight the effectiveness found in terms of variations, accuracy and tracking speed.
- Both techniques offer significant advantages for SWPS, providing a convincing solution for improving the overall performance and energy efficiency, although the parameters of each technique must be carefully selected according to the size of the installation in order to guarantee the good performances.

The remainder of this paper is organized as follows. Section 2 describes the modeling of this system, just as Section 3 explores the proposed optimization techniques Bat and DTC. Section 4 deals with a parametric study, analyzing the results and their implications. Finally, Section 5 summarizes the results and outlines prospects for future research.

## 2. Solar water pumping system modeling

**Fig 1:**
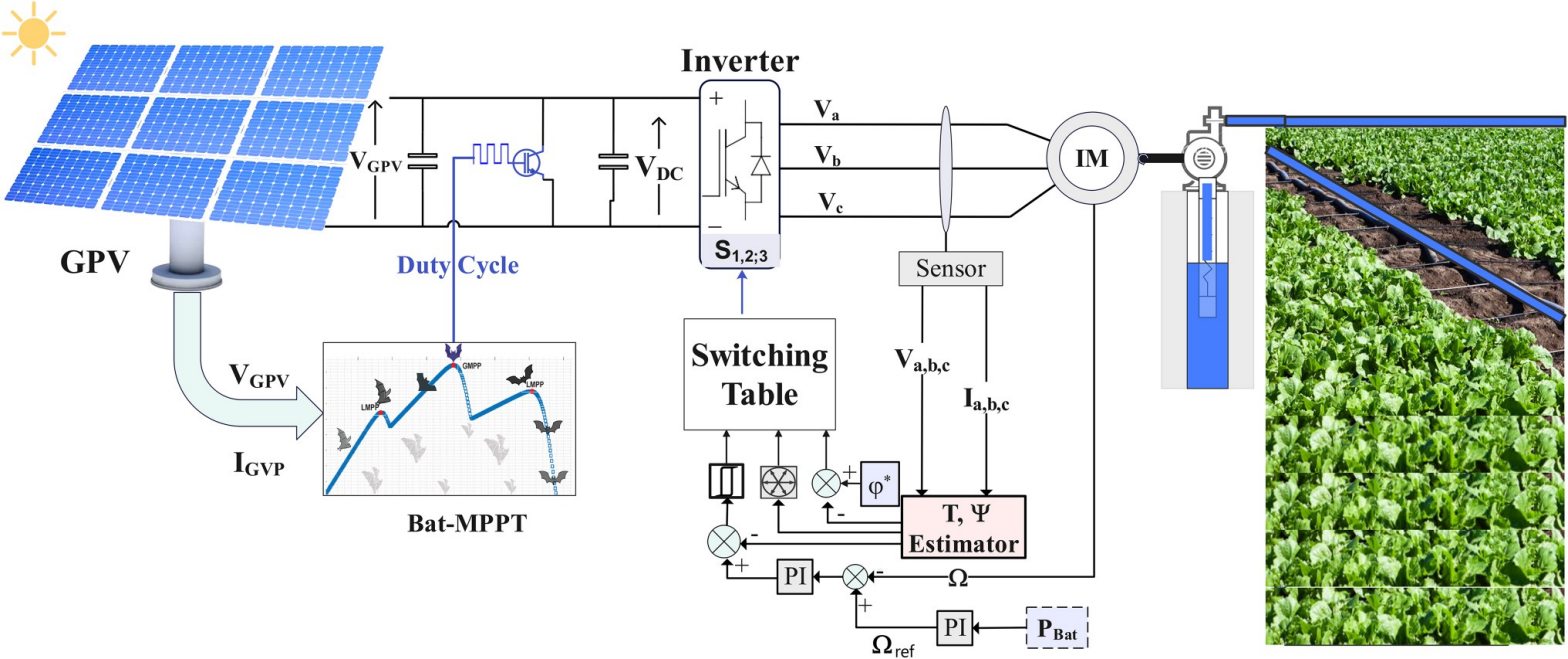
SWPS description diagram.

This section is devoted to modeling the different components of the solar water pumping system under investigation, which is illustrated in full in Fig 1. At the heart of the system is the photovoltaic generator (PVG), responsible for converting solar energy into electricity to power the motor-pump assembly. To improve the efficiency of energy production, the use of a boost converter regulates the duty cycle using a metaheuristic method based on the bat principle. This Bat optimization technique is used to accurately monitor the amount of energy available in the PVG in real time. The inverter, guided by the DTC control, adjusts the energy of the asynchronous machine according to the amount of energy available at the output of the DC-DC converter in an optimal way. The asynchronous motor, because of its robustness and ability to operate efficiently in a wide range of environments, turns the centrifugal pump to transport water to agricultural areas. The synergy of these components contributes to the overall performance of the solar water pumping system, ensuring efficient use of solar energy in the context of sustainable agricultural irrigation.

### 2.1 PVG for solar water pumping system

**Fig 2:**
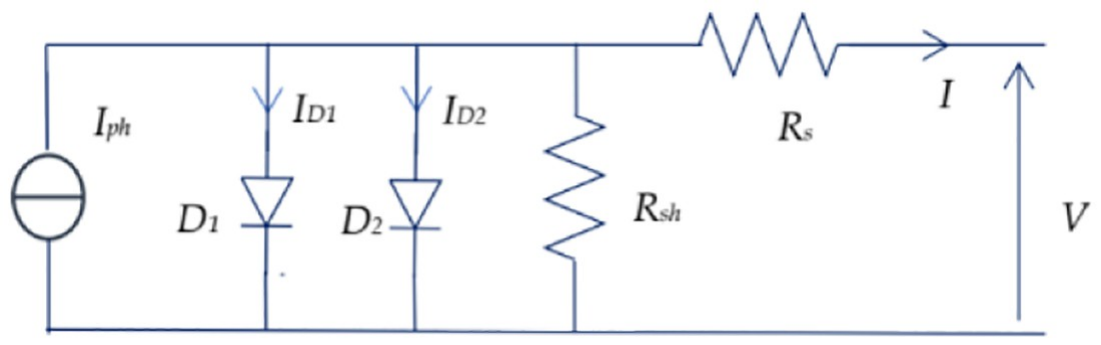
Two-diode electrical model of a photovoltaic cell.

Fig 2 depicts a two-diode electrical model [36]. This is the key element in in-depth studies of solar cell behavior within photovoltaic modules. The PV cells as the fundamental elements of these modules are often arranged in series and in parallel to create photovoltaic generators capable of producing the necessary voltage and current. In each photovoltaic module, several cells are carefully assembled, establishing series and parallel connections. This complex configuration meets the system’s need for sufficient electrical energy. Modeling solar cells is a delicate process, involving the choice of an appropriate model that may comprise one, two or even three diodes. This choice depends closely on the specific characteristics of the cells, such as the type of material used and the percentages of losses modeled by resistance and precision as a function of climate change.

The current-voltage characteristics of a photovoltaic cell are expressed in Eq (1) [37], where the thermodynamic potential (*V*_*th*_) is determined by key factors such as temperature, the Boltzmann constant (*K*), the electron charge (*e*), and the junction non-ideality factor (*n*_*1*_, *n*_*2*_). Temperature has a significant influence on the thermodynamic potential, while the Boltzmann constant (*K*) provides adjustments related to the energy distribution. The electron charge (*e*) is a fundamental component, and the non-ideality factor of the junction (*n*_*1*_, *n*_*2*_) introduces realistic nuances. These elements interact in a complex way to give the relationship between current (*I*) and voltage (*V*) at the cell output, which makes it possible to define the electrical performance of the photovoltaic cell under given conditions.


$$I=I_{ph}-I_{D1}\left[exp^{\frac{V+I.Rs}{n_{1}V_{th}N_{s}}}-1\right]-\frac{V+I.R_{s}}{R_{sh}}-I_{D2}\left[exp^{\frac{V+I.Rs}{n_{2}V_{th}N_{s}}}-1\right]-\frac{1}{R_{sh}}(IR_{s}+V)$$ (1)

Table 1 shows the various parameters of the main electrical characteristics inherent in each module studied. This is a Centrosolar America EP7 295 SW. The generator has five modules in series and one string in parallel.

**Table 1:** EP7 295 SW parameters.

*T*_*Voc*_	*T*_*Isc*_	*V*_*mpp*_	*I*_*mpp*_	*P*_*mpp*_	*I*_*sc*_	*V*_*oc*_
-0.0334 /deg.C	0.0655%/deg.C	35.47 V	8.32 A	295 W_c_	8.95A	46.2V

**Fig 3:**
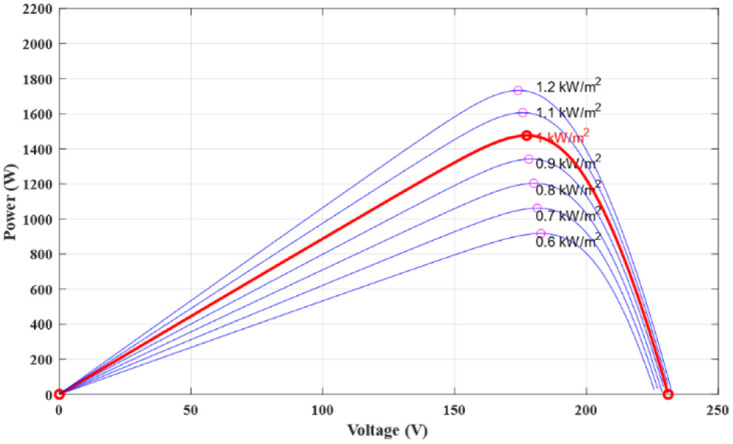
PVG Power characteristics under various irradiance.

Fig 3 completes the picture by highlighting the variation of the photovoltaic generator’s output in relation to irradiation fluctuations. This graphical representation clearly and concisely visualizes the direct impact of irradiation on the energy performance of the photovoltaic array, providing valuable information for the optimization and efficient management of solar power generation.

### 2.2 MPPT for solar water pumping system

**Fig 4:**
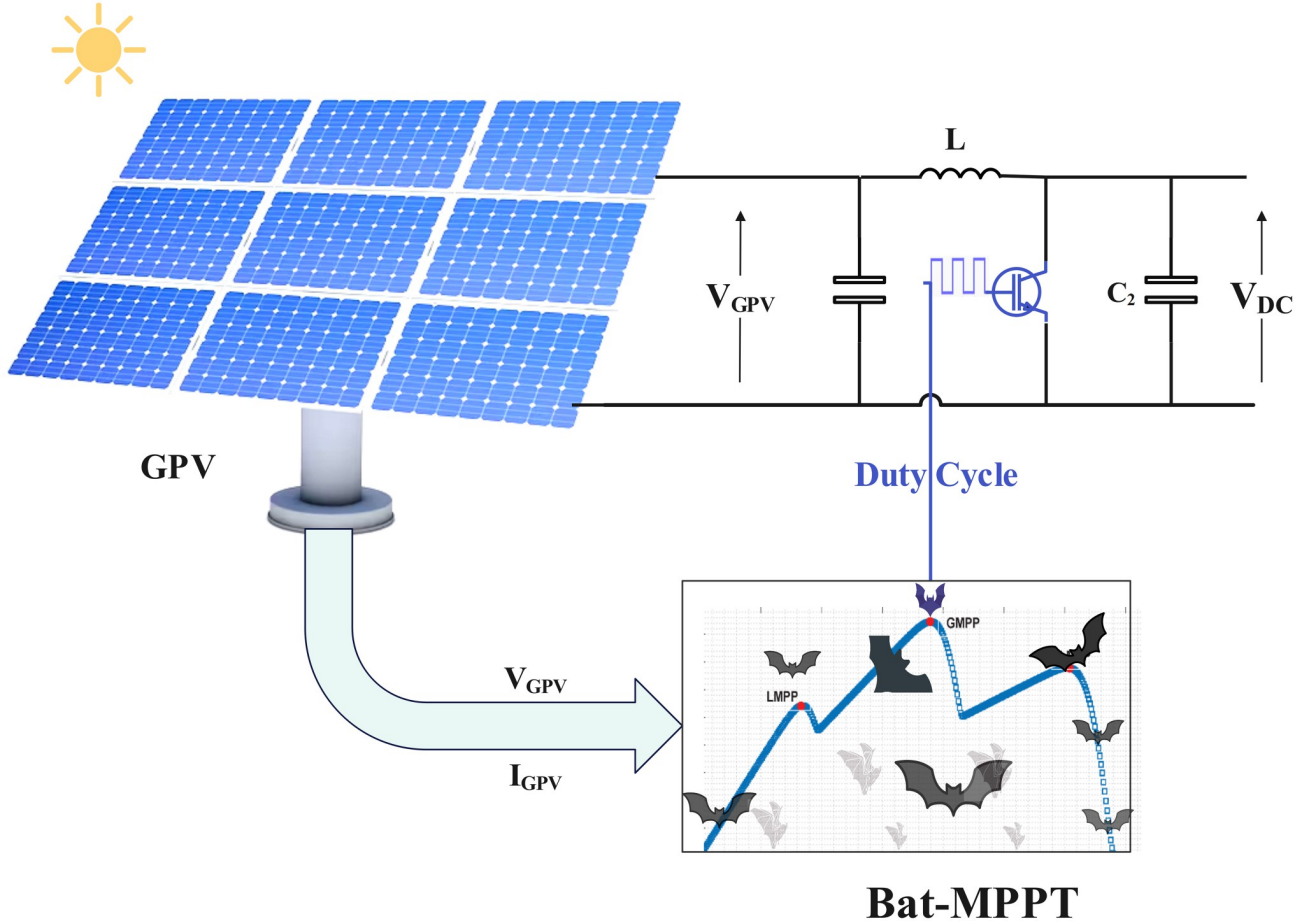
MPPT-bat for solar water pumping system.

The maximum power point tracker is an essential component of the solar water pumping system, designed as a boost converter composed of a inductor, a power switch and two filter capacitors, as shown in Fig 4. Its basic function is to regulate the generator output voltage by adjusting the duty cycle, using the *MPPT-Bat* technique in order to obtain the maximum power voltage. This process is designed to optimize energy transfer from the PVG to the centrifugal pump via the inverter, thereby maximizing the energy efficiency of the solar water pumping system. Eq (2) [38] describes the relationship between the input/output voltage of this tracker as a function of the duty cycle.


$$V_{DC}=\frac{V_{GPV}}{1-\alpha }$$ (2)

The Table 2 shows the different parameters of boost converter used in this investigation.

**Table 2:** Parameters of boost converter.

*Variable*	Value
*C1*, *C2*	*1mF*
*L*	3.7mH

### 2.3 Voltage inverter for solar water pumping system

**Fig 5:**
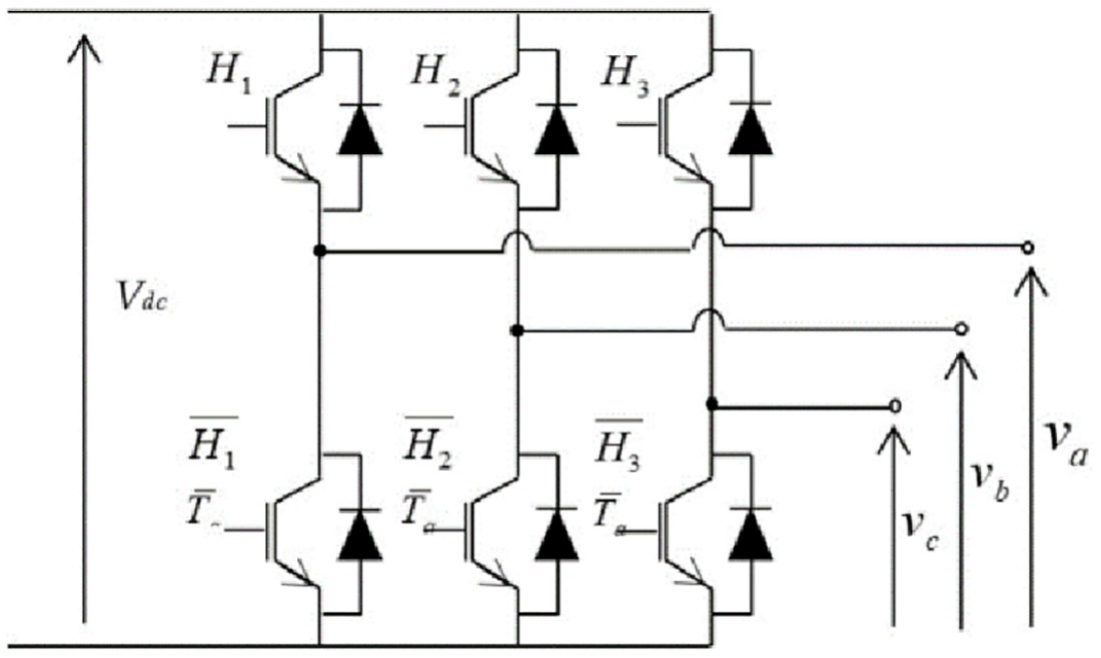
Structure of a two-stage voltage inverter for SWPS.

The voltage inverter is a static converter used to convert the DC bus voltage at the output of the boost converter into an AC voltage to power the induction motor employed to turn the pump. It is used to impose variable amplitude and frequency voltages on the motor in relation to the control commands for this solar water pumping system. The schematic diagram of this two-level voltage converter is shown in the Fig 5.

The architecture of this converter includes three independent arms, each equipped with two power switches. Each power switch is designed with a thyristor or a transistor associated with a diode in antiparallel. This switch must be equipped with a control circuit and a cooling block. The pairs of switches must be controlled in a complimentary way for the good of the installation. The state of the switches is defined by three Boolean control variables *H*_*i*_ *(i = 1*,*2*,*3)*. These will also be determined by the DTC control strategy described in this article. The conversion matrix used to model this type of energy transfer is given by Eq (3), where the simple voltages delivered by the inverter are obtained directly from the states of the control variables *H*_*1*,*2*,*3*_, which represent the control signals.


$$\left[\begin{array}{c} v_{a}\\ v_{b}\\ v_{c} \end{array}\right]=\frac{1}{3}V_{DC}\left[\begin{array}{ccc} 2 & -1 & -1\\ -1 & 2 & -1\\ -1 & -1 & 2 \end{array}\right]\left[\begin{array}{c} H_{1}\\ H_{2}\\ H_{3} \end{array}\right]$$ (3)

### 2.4 Asynchronous motor for solar water pumping system

The induction motor takes a leading role in the energy chain of this solar water pumping system. Its role is crucial, as it acts as a connecting link between the inverter, responsible for converting the energy extracted from the MPPT-PSO available in the DC bus into AC energy, and the centrifugal pump that efficiently extracts the water from its source. This remarkable relationship between the asynchronous motor and the system components generates a continuous flow of water, guaranteeing a reliable and constant supply to meet agricultural needs. It is therefore of the utmost importance to establish a formalized understanding of the behavior of the asynchronous motor. This can be achieved by describing its mathematical model in the reference frame (α, β), taking into account the many parameters. This model reduces the complexity of the machine’s three-phase representation (a,b,c), and it is expressed by the following equations [39].

- Electrical equations:
$$\left\{\begin{array}{l} v_{s\alpha }=R_{s}.i_{s\alpha }+\frac{d\psi_{s\alpha }}{dt}\\ v_{s\beta }=R_{s}.i_{s\beta }+\frac{d\psi_{s\beta }}{dt}\\ v_{r\alpha }=R_{r}.i_{r\alpha }+\frac{d\psi_{r\alpha }}{dt}+\omega_{m}.\psi_{r\beta }\\ v_{r\beta }=R_{r}.i_{r\beta }+\frac{d\psi_{r\beta }}{dt}-\omega_{m}.\psi_{r\alpha } \end{array}\right.$$ (4)
- Magnetic equations:
$$\left\{\begin{array}{c} \psi_{s\alpha }=L_{s}i_{s\alpha }+M.i_{r\alpha }\\ \begin{array}{l} \psi_{s\beta }=L_{s}i_{s\beta }+M.i_{r\beta }\\ \psi_{r\alpha }=L_{r}i_{r\alpha }+M.i_{s\alpha }\\ \psi_{r\beta }=L_{r}i_{r\beta }+M.i_{s\beta } \end{array} \end{array}\right.$$ (5)
- Mechanical equations:
$$T_{em}=p.(\psi_{s\alpha }i_{s\beta }-\psi_{s\beta }i_{s\alpha })$$ (6)
$$J.\frac{d\Omega }{dt}+f.\Omega =T_{em}-T_{r}$$
- The expression for electromagnetic torque is given by the following equation:
$$T_{em}=p.(\psi_{s\alpha }.i_{s\beta }-\psi_{s\beta }.i_{s\alpha })$$ (7)

The asynchronous motor draws its essence from these different principles, which can be presented in matrix format by Eq 8. This holistic approach provides a comprehensive understanding of the nuances involved in asynchronous motor operation, facilitating efficient use and optimization of the entire system.

$$\frac{d}{dt}\left[\begin{array}{l} i_{s\alpha }\\ i_{s\beta }\\ \psi_{s\alpha }\\ \psi_{s\beta } \end{array}\right]=\left[\begin{array}{cc} \begin{array}{c} -\frac{1}{\sigma }\left(\frac{1}{\tau_{s}}+\frac{1}{\tau_{r}}\right)\\ \begin{array}{c} \omega_{r}\\ \begin{array}{c} -R_{s}\\ 0 \end{array} \end{array} \end{array} & \begin{array}{cc} \begin{array}{c} -\omega_{r}\\ \begin{array}{c} -\frac{1}{\sigma }\left(\frac{1}{\tau_{s}}+\frac{1}{\tau_{r}}\right)\\ \begin{array}{c} 0\\ -R_{s} \end{array} \end{array} \end{array} & \begin{array}{cc} \begin{array}{c} \frac{1}{\sigma L_{s}\tau_{r}}\\ \begin{array}{c} -\frac{\omega_{r}}{\sigma L_{s}}\\ \begin{array}{c} 0\\ 0 \end{array} \end{array} \end{array} & \begin{array}{c} \begin{array}{c} \begin{array}{c} \frac{\omega_{r}}{\sigma L_{s}}\\ \frac{1}{\sigma L_{s}\tau_{r}} \end{array}\\ 0 \end{array}\\ 0 \end{array} \end{array} \end{array} \end{array}\right].\left[\begin{array}{l} i_{s\alpha }\\ i_{s\beta }\\ \psi_{s\alpha }\\ \psi_{s\beta } \end{array}\right]+\left[\begin{array}{cc} \begin{array}{c} \frac{1}{\sigma L_{s}}\\ \begin{array}{c} 0\\ 1\\ 0 \end{array} \end{array} & \begin{array}{c} \begin{array}{c} \begin{array}{c} 0\\ \frac{1}{\sigma L_{s}} \end{array}\\ 0 \end{array}\\ 1 \end{array} \end{array}\right].\left[\begin{array}{l} V_{s\alpha }\\ V_{s\beta } \end{array}\right]$$ (8)

with, $\sigma =1-\frac{M^{2}}{L_{s}L_{r}}$, $\tau_{s}=\frac{R_{s}}{L_{s}}$,$\tau_{r}=\frac{R_{r}}{L_{r}}$.

The Table 3 shows the different parameters of the asynchronous motor used in this investigation.

**Table 3:** Parameters of induction motor.

*Variable*	Value
*Pair pole number*	*4*
*Stator resistance*	*R*_*s*_ = 6.751 Ω
*Rotor resistance*	*R*_*r*_ = 6.215 Ω
*Mutual inductance*	L_m_ = 0.5 H
*Total inertia*	*J* = 0.0144 Kg.m²
*Self-inductances*	*L*_*s*_ = 0.52H; *L*_*r*_ = 0.52 H
*Viscous frictions*	*f* = 0.0020 Kg.m²/s

### 2.5 Centrifugal pump for solar water pumping system

The industry has made remarkable progress in developing various types of hydraulic pumps, such as positive displacement, piston and centrifugal models. The centrifugal pumps became the most appropriate choice for pumping applications [40]. This is because the resistive torque generated by the centrifugal pump is ingeniously formulated to respect the quadratic structure of the drive speed, as elegantly demonstrated by Eq (9). A careful analysis of the characteristics of the torque/speed relationship reveals the insignificance of the initial torque exerted on the pump. This characteristic can be attributed to the pump’s remarkable ability to operate optimally even at extremely low solar irradiation levels. As solar irradiation levels increase, the drive motor can reach higher speeds, inducing instantaneous flow.


$$T_{r}=\text{A}.\Omega^{2}$$ (9)

$$Q_{2}=\frac{\Omega_{1}}{\Omega_{2}}.Q_{1}$$ (10)

## 3. Solar water pumping system optimization

This section is devoted to the two approaches used to optimize solar water pumping systems. First, the MPPT metaheuristic technique based on the Bat principle. The main objective is to maximize the extraction of available power from the PVG. This intelligent approach adjusts the electrical parameters of the solar water pumping system in real time to ensure that the PVG is constantly operating at the MPP. Furthermore, particular attention focuses on the DTC technique applied to the induction motor side of the system. This approach aims to make more efficient use of the power extracted from the solar generator. By regulating the torque and magnetic flux of the induction motor directly and precisely. This dual approach, combining MPPT to optimize solar extraction and DTC for efficient generator operation, is a powerful strategy for improving the reliability and overall efficiency of solar water pumping systems.

### 3.1 Optimization technique based on the Bat principle

The bat technique is a metaheuristic optimization method inspired by nature. This technique is used to improve the energy efficiency of a photovoltaic system. In this context, the main objective is to adjust the PV generator’s electrical parameters, such as operating voltage and current, in order to maximize the power output of PVGs [41]. The bat metaheuristic method aims to find efficient solutions without the need for testing, training or non-linear modeling of the PVGs. It focuses on optimizing the electrical parameters PVGs without requiring a precise model. The bat algorithm, by its iterative nature, constantly strives to find the optimal maximum power point, reacting to changing environmental conditions.

The first step consists of initializing the photovoltaic system, where the specific parameters of the PVG are defined, such as maximum operating voltage, short-circuit current, and rated power. In addition, the parameters of the bat algorithm are also initialized, such as the number of bats, the pulse frequency, the pulse amplitude, and the search bounds for the PV generator parameters. At the start of the optimization, the chopper duty cycle is initialized to 0.5, which assumes that the operating point of the pumping system lies at the intersection of the current-voltage characteristics of the PVG and the torque imposed by the induction motor.

Once the system is configured, the main loop of the bat algorithm begins. Bats position themselves randomly (*x*_*i*_) and fly randomly with fixed velocity (*v*_*i*_) and frequency (*f*), respectively according to Eqs (12–13) [42], and vary wavelengths when foraging [43]. At each iteration, the bats adjust their frequencies by a random vector drawn from a uniform distribution that corresponds to the electrical voltage of PVGs, thereby adjusting their positions according to Eq (11). This generally involves modifying the operating voltage to get closer to the global power point.


$$x_{i}^{t+1}=x_{i}^{t}+v_{i}^{t+1}$$ (11)

$$v_{i}^{t+1}=v_{i}^{t}+\left(x_{i}^{t}-x^{*}\right)f_{i}$$ (12)

$$f_{i}=f_{min}+(f_{max}-f_{min})\beta$$ (13)

Once the various outputs have been calculated, the algorithm compares these values to find the optimum output, which corresponds to the maximum overall power the PVG can deliver under the given operating conditions. To do this, it calculates the errors between the different power ratings and selects the best solution that optimizes overall system performance.

**Fig 6:**
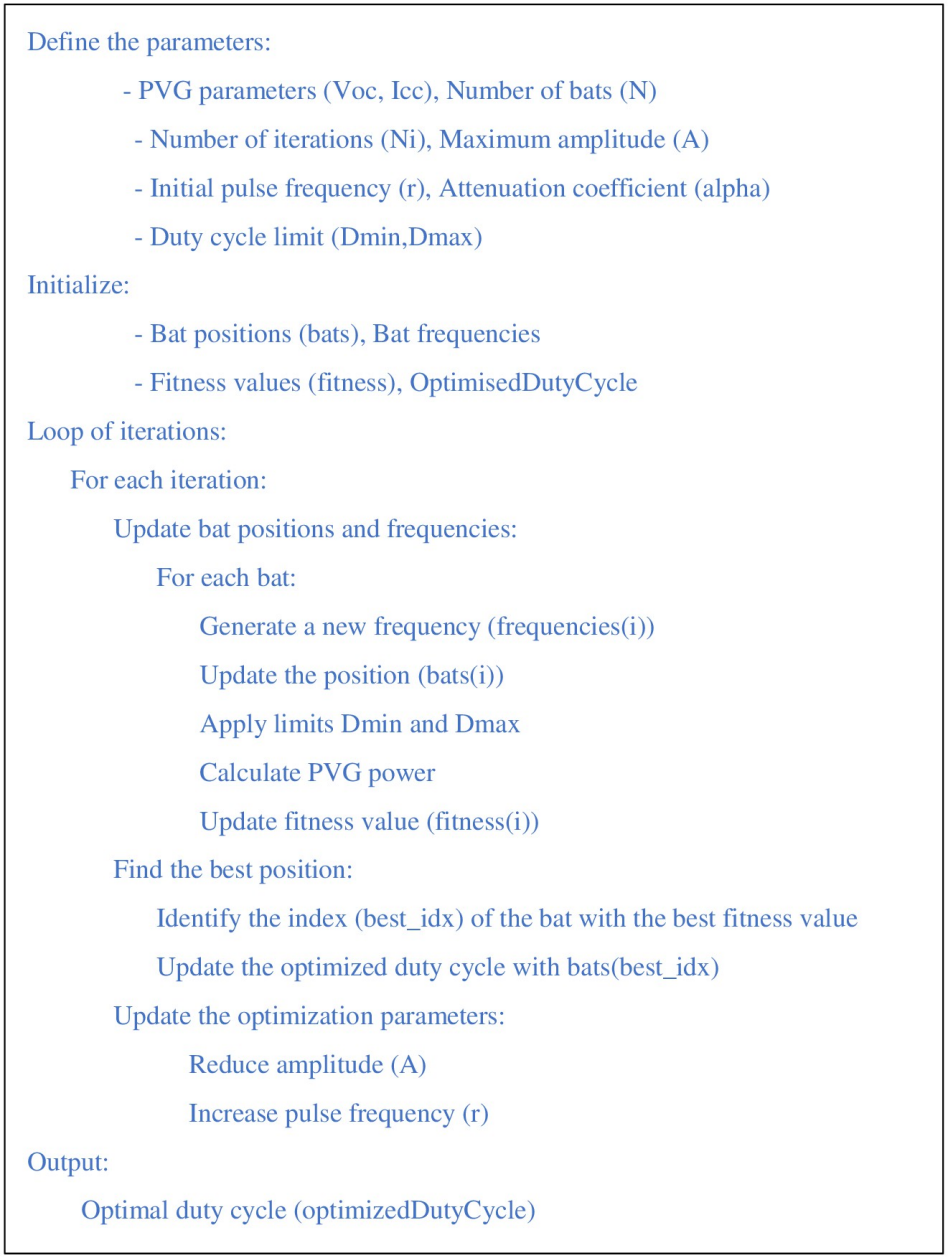
Pseudocode for MPPT-bat technique.

System performance is assessed by calculating the power output of the PVG parameter set, using the current irradiation conditions. The process continues by updating the best set of local and global parameters based on the calculated performance of each individual. The aim is to identify the parameters that maximize energy production. The tracking error is calculated by subtracting the measured current power of the solar array with the best overall power found. This error represents the gap between the PV generator’s current performance and its optimum potential. Finally, the PVG parameters are adjusted according to this tracking error, enabling the system to converge towards the MPP. At the end of the loop, once predefined stopping criteria has been reached (such as a specific number of iterations), the optimal electrical parameters of the PVG are determined [44]. These parameters aim to maximize the solar panel’s power output under the environmental conditions. The stages of this process are described in detail in the diagram in Fig 6. The parameters used to optimize the pumping system are shown in Table 4.

**Table 4:** various parameters of MPPT-Bat.

*V*_*oc*_	*I*_*cc*_	*N*	*alpha*	*D*_*min*_	*A*	*Ni*	*D*_*max*_	*f*_*i*_
**240 V**	11 A	45	0.67	0.1	0.5	70	0.6	0.1

### 3.2 DTC techniques for solar water pumping systems

**Fig 7:**
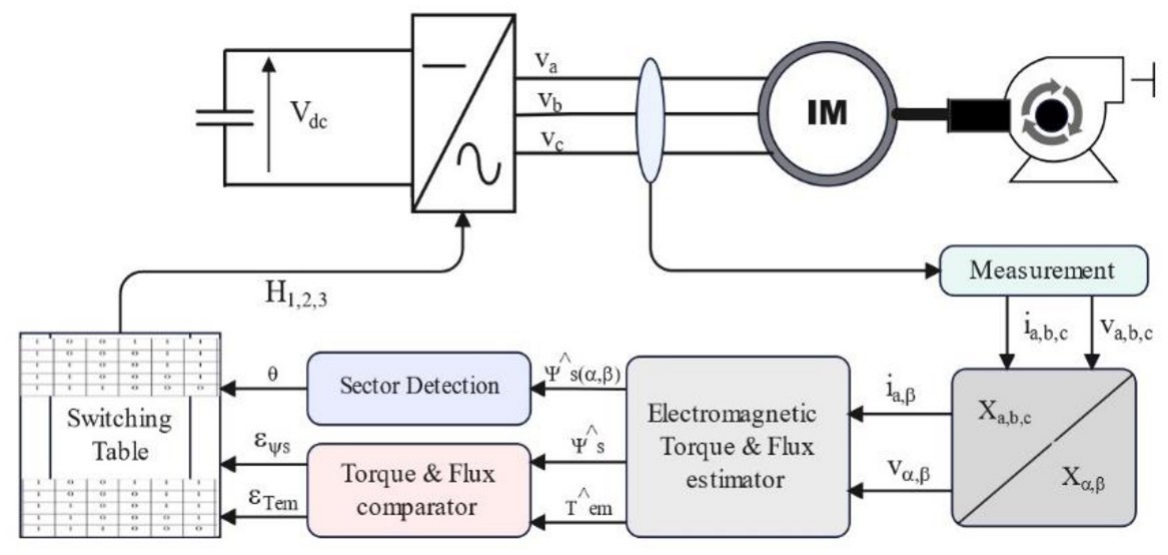
DTC control principle applied to the solar water pumping system.

The control strategy used to drive the induction machine is based on Direct Torque Control. This approach is chosen to optimally exploit the energy generated by the MPPT, which is based on the bat metaheuristic technique. The fundamental objective of DTC is to continuously adjust stator energy flow and electromagnetic force by implementing hysteresis controllers and a switching table to control the inverter, as shown in Fig 7.

To simplify the study of the time equations, a transformation is performed on the three-phase stator field windings, converting them into fictitious two-phase windings in the (α, β) reference frame using the Concordia transformation, the calculation procedure which is defined by Eq (14) [45]. This transformation facilitates the estimation of stator flux and electromagnetic torque. The components (α, β) of the stator currents and voltages are used to calculate the stator flux and electromagnetic torque, as detailed later. This approach offers a more practical representation of the relevant variables in this repository for better management of the induction machine control system.


$$\left[\begin{array}{l} X_{\alpha }\\ X_{\beta } \end{array}\right]=\sqrt{\frac{2}{3}}.\left[\begin{array}{cc} \begin{array}{c} \begin{array}{l} 1 \end{array}\\ 0 \end{array} & \begin{array}{cc} \begin{array}{c} -\frac{1}{2}\\ \frac{\sqrt{3}}{2} \end{array} & \begin{array}{c} -\frac{1}{2}\\ -\frac{\sqrt{3}}{2} \end{array} \end{array} \end{array}\right].\left[\begin{array}{l} X_{a}\\ X_{b}\\ X_{c} \end{array}\right]$$ (14)

#### a. Stator flux estimation

The Stator flux is estimated from stator voltage and current vectors, expressed by Eq (15).


$${\bar{\psi }}_{s}(t)=\int_{0}^{t}({\bar{V}}_{s}+R_{s}.{\bar{I}}_{s}).dt$$ (15)

The stator flux vector is calculated from its two-phase components of axes (α,β), as in Eq (16).


$${\bar{\psi }}_{s}=\psi_{s\alpha }+j.\psi_{s\beta }$$ (16)

The stator flux modulus is written as:

$${\hat{\psi }}_{s}=\sqrt{{\hat{\psi }}_{s\alpha }^{2}+{\hat{\psi }}_{s\beta }^{2}}$$ (17)

With,

$$\begin{array}{cc} {\hat{\psi }}_{s\alpha }=\overset{t}{\underset{0}{\int }}\left(v_{s\alpha }-R_{s}.i_{s\alpha }\right).dt & {\hat{\psi }}_{s\beta }=\overset{t}{\underset{0}{\int }}\left(v_{s\beta }-R_{s}.i_{s\beta }\right).dt \end{array}$$ (18)

The stator current components are obtained by applying the Concordia transformation to the measured currents.


$$\left\{\begin{array}{c} i_{s\alpha }=\sqrt{\frac{3}{2}}.i_{sa}\\ i_{s\beta }=\frac{1}{\sqrt{2}}.(i_{sb}-i_{sc}) \end{array}\right.$$ (19)

The components of the stator voltage vector *v*_*sα*_ and *v*_*sβ*_ are obtained from the measurement of the *DC* voltage *V*_*dc*_ and the state of switches *H*_*1*,*2*,*3*_, using Eq (20).


$$\left\{\begin{array}{c} v_{s\alpha }=\sqrt{\frac{3}{2}}.V_{dc}.\left[H_{1}-(H_{2}+H_{3})\right]\\ v_{s\beta }=\frac{1}{\sqrt{2}}.V_{dc}.(H_{2}-H_{3}) \end{array}\right.$$ (20)

The sector *S*_*i*_ in which the stator flux vector lies is determined by the components *ψ*_*sα*_ and *ψ*_*sβ*_. The angle *θ*_*s*_ between the stator frame of reference and the vector ${\vec{\text{ψ}}}_{s}$, calculated by Eq (21).


$$\theta_{s}=arctg(\frac{{\hat{\psi }}_{s\beta }}{{\hat{\psi }}_{s\alpha }})$$ (21)

#### b. Electromagnetic torque estimation

The electromagnetic torque can be estimated from the estimated fluxes and measured currents, and expressed in the form of Eq (22). From this equation, it can be seen that the accuracy of the electromagnetic torque modulus depends on the accuracy of the stator current measurement and the quality of the stator flux estimation.


$${\hat{T}}_{em}=p.({\hat{\psi }}_{s\alpha }.i_{s\beta }-{\hat{\psi }}_{s\beta }.i_{s\alpha })$$ (22)

#### c. Flux controller design

**Fig 8:**
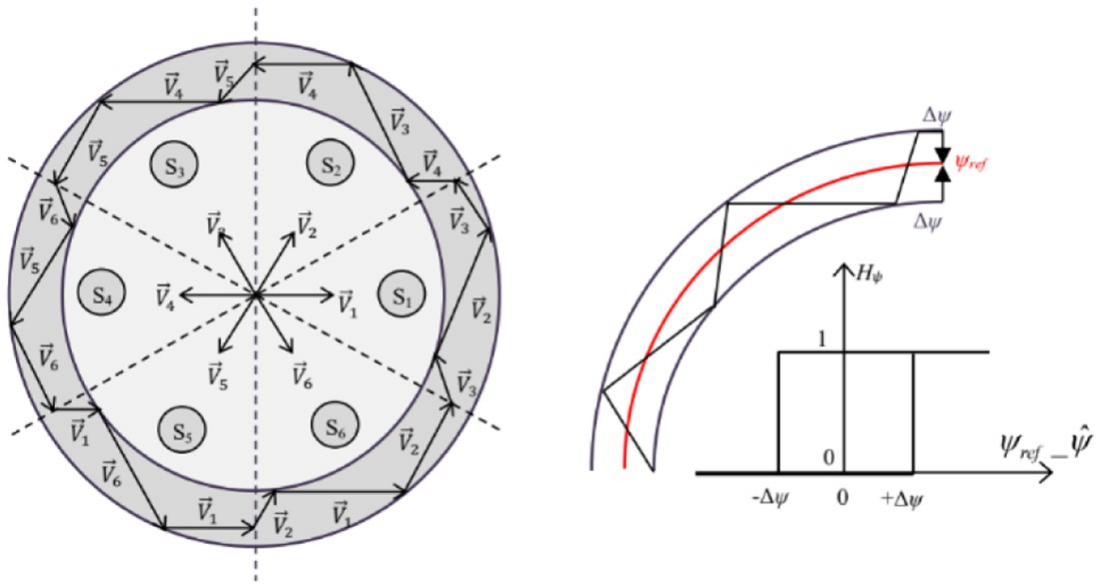
Hysteresis comparator principle for stator flux control.

This corrector is designed to keep the end of the flux vector within a circular band, as shown in the Fig 8. A hysteresis comparator is used for stator flux correction. The comparator output indicates the direction of flux modulus evolution, in order to select the appropriate voltage vector. The difference between the reference flux and the estimated flux is entered into a two-level hysteresis controller, which generates the Boolean variable *Hψ* at its output [46]. This variable directly indicates whether the flux amplitude should be increased *(Hψ = 1)* or decreased *(Hψ = 0)* in order to keep the difference between the estimated flux and the flux reference within a hysteresis width *Δψ*, and the inequation $\left|\varepsilon =\psi_{ref}\_\hat{\psi }\right|\leq \Delta \psi$ should be checked. If *ε > Δψ* or *ε ˂- Δψ*, this means that the flux is moving out of the hysteresis band. In the first case, we’ll need to impose a voltage vector that will increase the flux modulus. In the second case, we’ll impose a voltage vector whose effect will be to decrease the flux modulus.

#### d. Torque controller design

**Fig 9:**
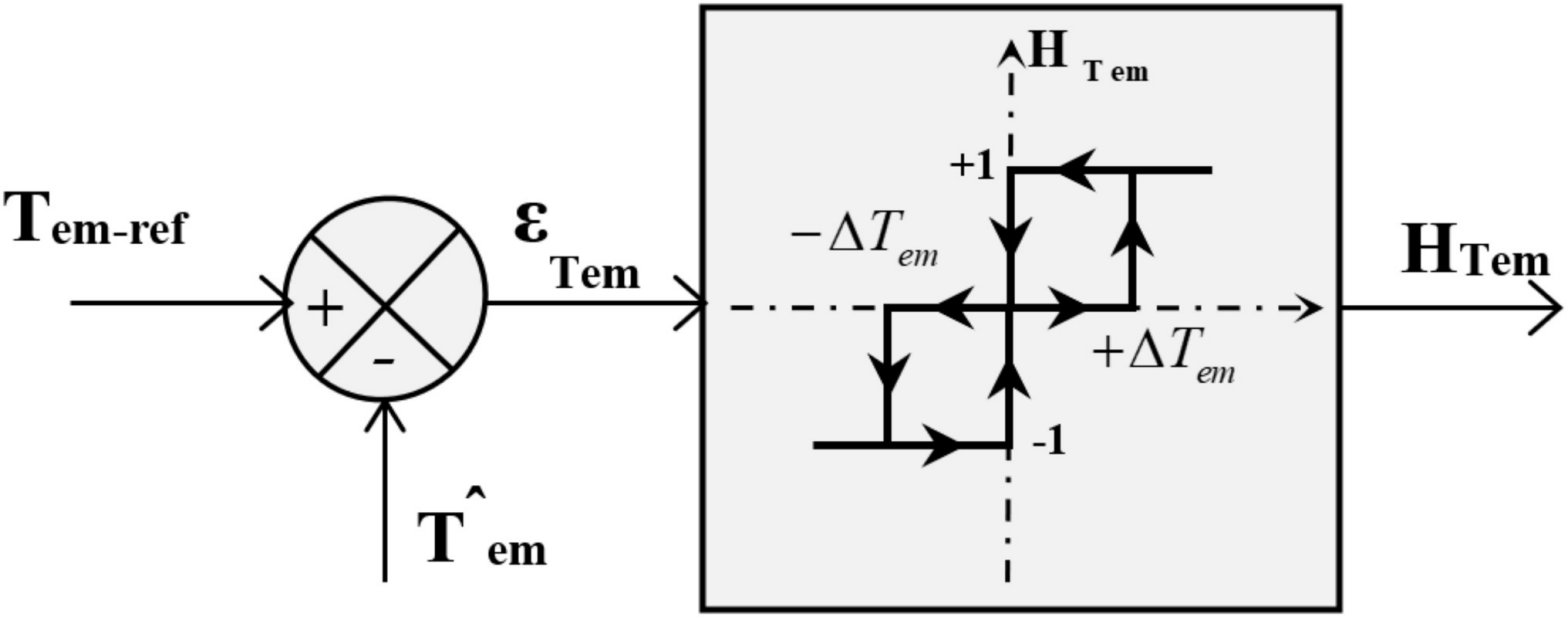
Electromagnetic torque hysteresis controller.

The principle of this hysteresis corrector is illustrated in Fig 9. Its role is to maintain the electromagnetic torque within the difference between the reference torque and the estimated torque within a hysteresis band. This difference is fed into a three-level hysteresis comparator, which generates at its output a Boolean variable directly indicating whether the torque amplitude should be increased in absolute value (*H*_*Tem*_ *= 1* for a positive setpoint and *H*_*Tem*_ *= -1* for a negative setpoint) or decreased (*H*_*Tem*_ *= 0*). It should be noted that increasing the levels of the corrector minimizes the average switching frequency of the switches [46].

#### e. Switching table design

The two-level voltage inverter supplies the asynchronous motor via a control command generated from a switching table of this DTC controller. The latter selects the appropriate voltage vector at each sampling time according to the state of the Boolean variables (*Hψ & H*_*Tem*_) at the output of the two flux correctors and the electromagnetic torque, as well as the Si sector giving information on the position of the flux vector in the (*α*,*β*) space, which is summarized by the Table 5 [47].

**Table 5:** DTC controller switching table.

		*Secteur S*_*i*_					
*H*_*ψs*_	*H*_*Cem*_	*S*_*1*_ [-π/6,π/6]	*S*_*2*_ [π/6,3π/6]	*S*_*3*_ [3π/6,5π/6]	*S*_*4*_ [5π/6,7π/6]	*S*_*5*_ [7π/6,9π/6]	*S*_*60*_ [9π/6,11π/6]
*1*	*1*	*v*_*2*_	*v*_*3*_	*v*_*4*_	*v*_*5*_	*v*_*6*_	*v*_*1*_
	*0*	*v*_*7*_	*v*_*0*_	*v*_*7*_	*v*_*0*_	*v*_*7*_	*v*_*0*_
	*-1*	*v*_*6*_	*v*_*1*_	*v*_*2*_	*v*_*3*_	*v*_*4*_	*v*_*5*_
*0*	*1*	*v*_*3*_	*v*_*4*_	*v*_*5*_	*v*_*6*_	*v*_*1*_	*v*_*2*_
	*0*	*v*_*0*_	*v*_*7*_	*v*_*0*_	*v*_*7*_	*v*_*0*_	*v*_*7*_
	*-1*	*v*_*5*_	*v*_*6*_	*v*_*1*_	*v*_*2*_	*v*_*3*_	*v*_*4*_

## 4. Results and discussion

**Fig 10:**
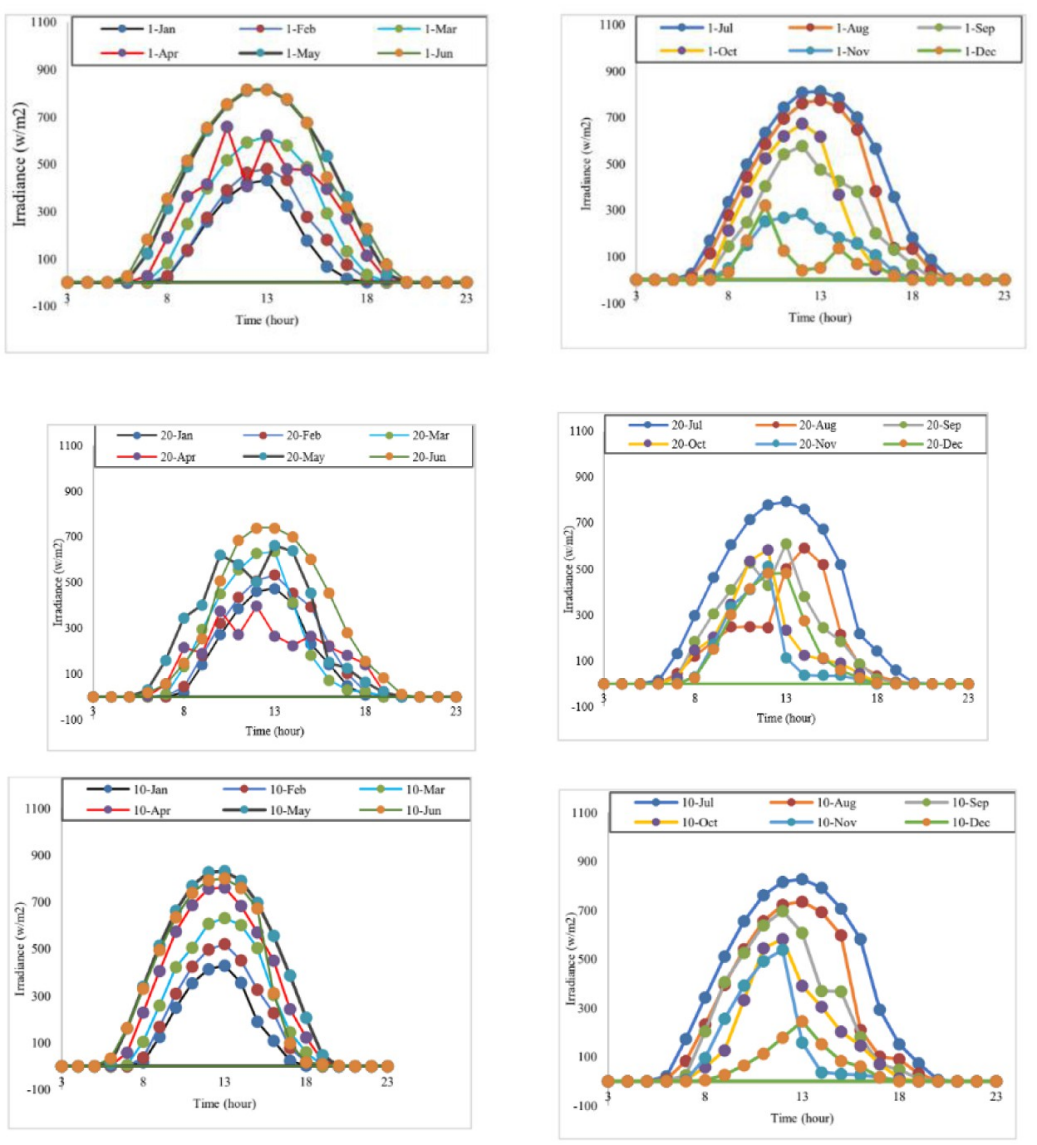
Average irradiance in the region in 2022.

The performance of the SWPS is related to the region’s solar deposit and its evolution in relation to other climatic changes such as temperature, humidity and especially irradiance, which is the most important and variable parameter during the day. In this contribution, we keep all parameters constant and test the robustness of the system as a function of irradiance, because the other parameters act with a very low correction coefficient, so that, for example, the module temperature studied affects power by 0.0334. However, as the performance is closely linked to the amount of irradiation available, it must be evaluated by carefully choosing the most appropriate form of evolution of this variable. This contribution is based on real irradiation data collected by a meteorological station at the Meknes Faculty of Science, recorded every hour during the year 2022. This station records minimum, average and maximum variations in several meteorological parameters such as temperature, wind speed, precipitation, irradiation and many other climatic parameters. The choice of setpoint for performance tests must be based on the evolution of real values; in this case, it is based on the analysis of the evolution of 36 days, randomly selected, corresponding to the first, tenth and twentieth days of each month, as illustrated in Fig 10. A detailed analysis of the data revealed different forms of irradiance profile during time intervals, such as uniform ("constant change"), abrupt ("rapid change") and constant ("constant value"). It should also be noted that the irradiation profile illustrated corresponds to the average irradiation; according to a detailed analysis of this variable by the monitoring station, its maximum variation can reach 250w.m^-2^ compared to the average irradiation. In addition, a maximum value of 1152w.m^-2^ was reached on Tuesday, May 03, 2022, in the region.

### 4.1 Irradiance profile used to assess the performances of SWPS

**Fig 11:**
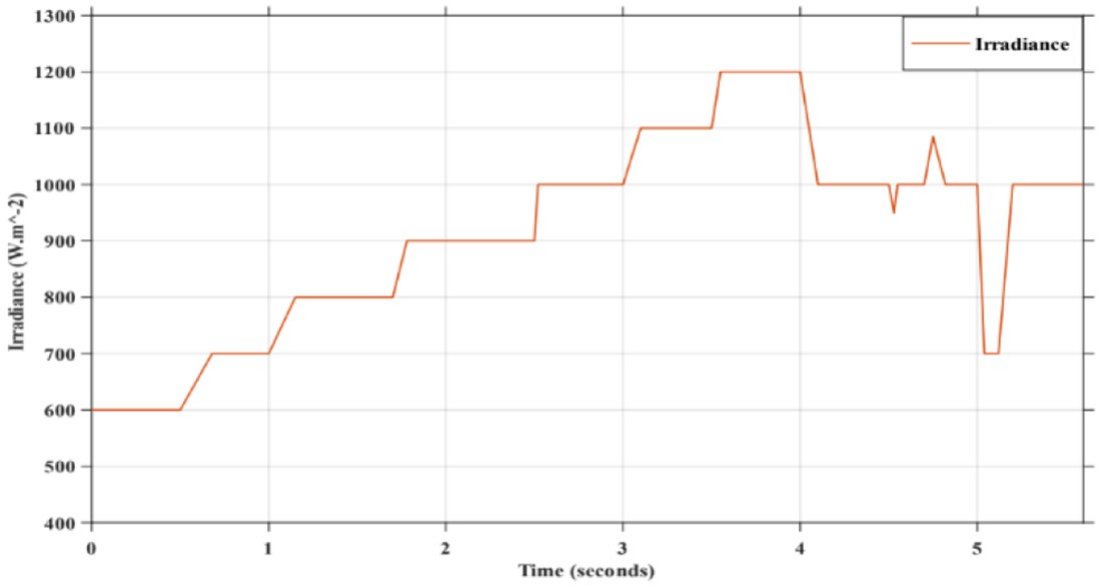
Irradiation profile used to assess performances SWPS.

The performance of the solar water pumping system is evaluated in this study using two different approaches. Firstly, the global power point tracking method using the bat metaheuristic technique on the PVG side. Secondly, the extracted energy is utilized by a DTC controller on the induction machine side. This evaluation is carried out under fluctuating irradiance conditions, simulating realistic situations. This analysis is based on the results shown in Fig 10. To test the robustness of the solar water pumping system, the profile used in Fig 11 is specially designed to include fast, slow, abrupt and habitual changes. These variations represent a remarkable test that provides an in-depth assessment of the system’s operation under diverse and dynamic conditions that reflect the reality of irradiance changes.

### 4.2 Simulation results

**Fig 12:**
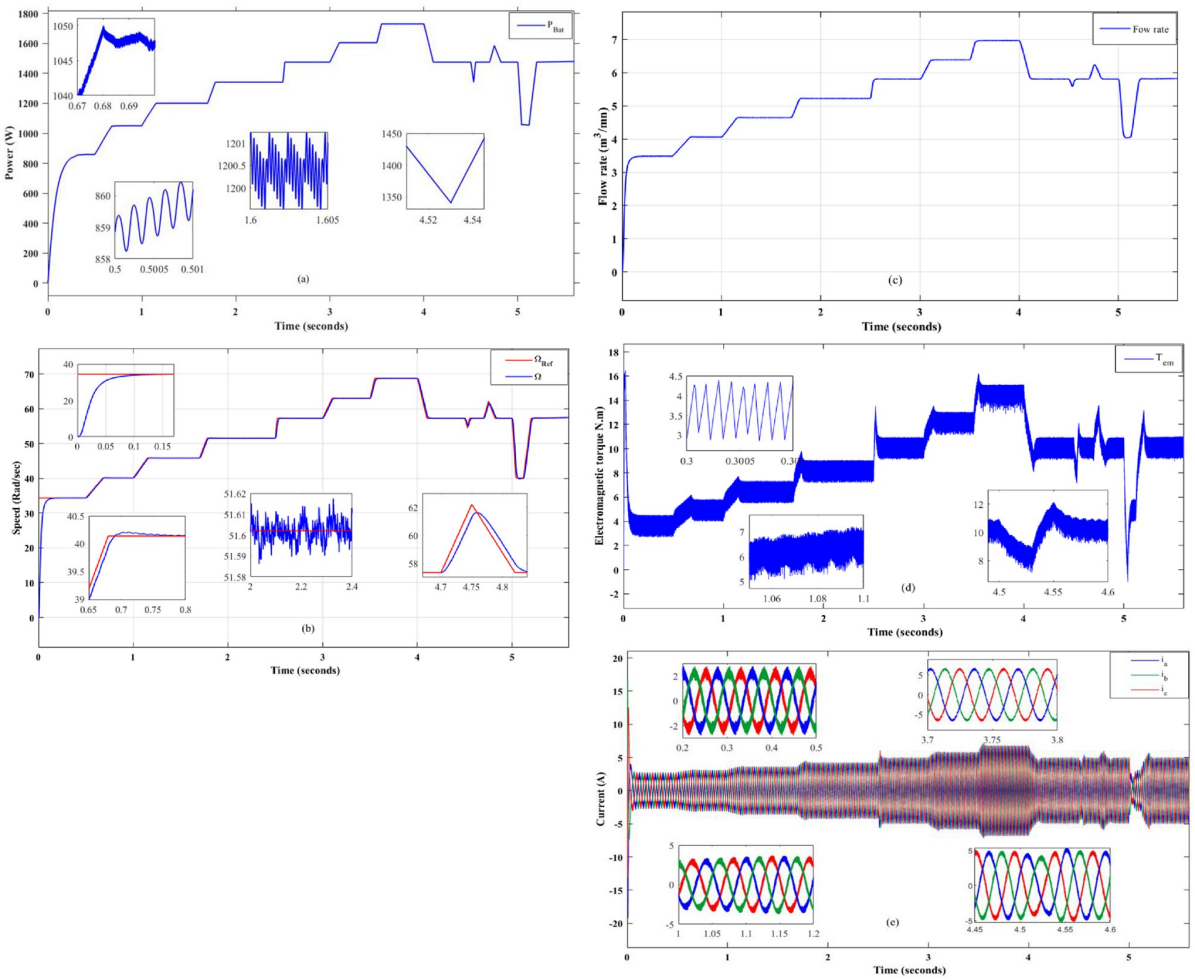
SWPS optimization results.

Fig 12 shows the evolution of the main electrical, mechanical and hydraulic parameters of the solar water pumping system, namely Fig 12(a) the power obtained from the photovoltaic generator using the MPPT-bat technique, Fig 12(b) the speed of the motor-pump unit using the DTC technique, Fig 12(c) the instantaneous flow rate as a function of time, Fig 12(d) the evolution of the electromagnetic torque of the induction machine and Fig 12(e) the current absorbed by the machine as a function of time. These quantities are used to evaluate the performance of the system.

### 4.3 Analysis and discussion

The integration of the MPPT-bat and DTC optimization techniques on this SWPS demonstrates a remarkable performance, as shown by the results in Fig 12. The advantages offered by these two techniques can be summarized as follows:

- The performance obtained using the MPPT-bat technique, shown in Fig 12(a), demonstrates a fast response of the system to different irradiance conditions. This responsiveness is interpreted by the increased efficiency of the proposed technique.
- The behavior of the induction machine speed in Fig 12(b) shows that the system responds quickly, accurately, without static or drag errors and with a low overshoot of less than 0.4%. This dynamic tracking confirms the success of the DTC control in ensuring system reliability.
- The evolution of the instantaneous flow rate is shown in Fig 12(c), which shows a similarity between the irradiance and the flow rate of the pumped water. This linearity is also justified by the relationship between flow rate and velocity in equation (10) and the results in Fig 12b. These results show the success of the two proposed techniques in power extraction and the application of compatible electromagnetic torque in Fig 12(d) for good energy management.
- Fig 12(e) shows the evolution of the current drawn by the induction motor. The sinusoidal shape of the absorbed current indicates a low level of harmonic distortion, which means a good quality of energy transfer to the induction machine. In addition, the evolution of the current is linearly related to the irradiance, further demonstrating the success of the two techniques proposed for the energy optimization of this SWPS.

To further validate the performance of the proposed techniques, Table 6 illustrates a comparative study between the maximum power that can be supplied by the PVG from Fig 3 and the power extracted using the MPPT-bat under different climatic conditions from Fig 12(a).

**Table 6:** Comparative study of power versus irradiance.

Irradiance (W.m^-2^)	PVG Power (W)	MPPT-Bat Power (W)	MPP Voltage (V)
700	*1051*	*1051*	*181*.*32*
800	*1200*	*1200*	*179*.*98*
900	*1341*	*1341*	*178*.*15*
1000	*1475*	*1475*	*177*.*35*
1100	*1606*	*1606*	*175*.*83*
1200	*1732*	*1732*	*174*.*07*

The results obtained highlight the positive performance of the solar water pumping system studied. The absence of static errors allows precise regulation of the irrigation flow rate, which can be particularly advantageous in slightly sloping terrain. These results suggest that the system has great potential for efficient use of water resources, contributing to increasing agricultural yields.

### 4.4 Results summary

In order to highlight the usefulness and success of the proposed technique, a comparative study with other techniques is presented in Table 7. This study examines performance on a global scale, as it’s difficult to find optimization techniques applied to the same water pumping system. This is due to the diversity of possibilities available for realizing such a solar pumping system. This diversity is due to the different types of static inverters (choppers, inverters), the different types of storage such as water tower or batteries, as well as the types of electric motors driving the hydraulic pump (DC, AC). It should also be noted that when the size of the system changes, parameters such as the sizing and choice of power components need to be adapted. This adaptation is essential to maintain system efficiency and reliability. The results of this comparative analysis underline the remarkable effectiveness of the proposed method, highlighting its relevance and potential in different application contexts.

**Table 7:** Comparative study of SWPS optimization techniques.

MPPT	Electric motor controller	Efficiency	Tracing speed	Implementation Complexity	Track Global MPP
**Kalman filter** [39]	*DTC*	*Good*	*Medium*	*Medium*	*No*
**FOCV** [48]	*Scalar control*	*Medium*	*Slow*	*Simple*	*No*
**FoFL** [49]	*-*	*Very Good*	*Fast*	*Complex*	*Yes*
**Neural network** [50]	*-*	*Very Good*	*Fast*	*Complex*	*Yes*
**CHHO** [51]	*DTC*	*Very Good*	*Fast*	*Complex*	*Yes*
**Proposed Bat**	*DTC*	*Very Good*	*Fast*	*Medium*	*Yes*

### 4.5 Validation with a real profile

This section uses an authentic setpoint data collected from a meteorological station located at the Faculty of Science in the city of Meknes, Morocco. As the solar water pumping system can only operate during periods of sunshine, the irradiation setpoint is used exclusively for a total duration of 16 hours, with the simulation starting at 6 a.m. The main objective is to carefully evaluate the effectiveness and efficiency of the two proposed techniques. These careful evaluations are intended to provide a clear understanding of the overall effectiveness of this approach. They are also intended to inspire and motivate farmers to adopt these highly beneficial systems to meet the needs of sustainable, environmentally friendly agriculture.

**Fig 13:**
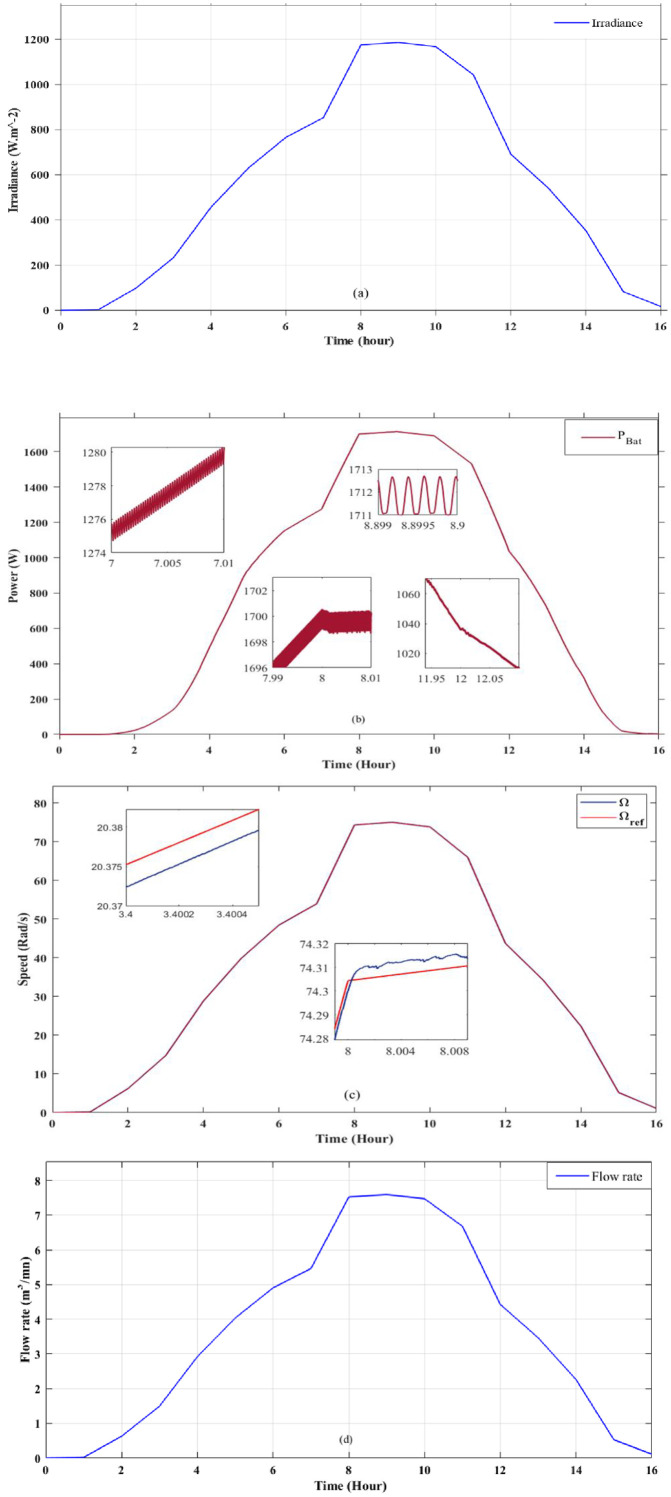
SWPS optimization results for the real setpoint.

The results show that both techniques perform exceptionally well, exceeding expectations. Bat’s optimization technique not only controls the maximum available power point in all cases, but also guarantees minimum disturbance, as shown visually in Fig 13(b). This level of control guarantees optimum energy extraction, thus optimizing the process. On the other hand, Direct Torque Control (DTC) ensures that the system perfectly maintains the predetermined speed setpoint throughout the day. The precision with which it maintains the pump speed, as clearly shown in Fig 13(c), guarantees a stable, optimum water flow, as shown in Fig 13(d). These results hold great promise for improving the energy efficiency of water pumps, as they show that the combination of DTC and Bat techniques can effectively overcome the limitations of conventional maximum power point tracking approaches. As a result, this combination offers a number of benefits that can be summarized as follows: First and foremost, it leads to an increase in solar power generation, thereby reducing costs and strengthening sustainability efforts. It also improves the quality of the power supply, extending the life of the system and minimizing the risk of operational disruption. In addition, the fusion improves the tracking speed, which ultimately increases the flow rate of the pumped water. Finally, it helps to reduce the carbon footprint, which plays an important role in preserving the environment. Taken together, these benefits offer a wealth of promising prospects for the integration of solar water pumping systems into sustainable agriculture.

## 5. Conclusion

This research paper investigates an approach to SWPS optimization. The approach is based on the use of MPPT-bat techniques on the PVG side and DTC on the induction motor side. These techniques respond quickly to irradiance changes, ensure that the pump motor speed is maintained accurately and establish an effective correlation between the instantaneous water flow rate and the amount of energy available in the PVG. The implications of these results are very encouraging as they have the potential to improve the energy efficiency of renewable SWPS. One of the key benefits of MPPT-bat is its ability to extract maximum power from the PVG regardless of weather conditions. In addition, the DTC enables precise, responsive control of the pump motor, optimizing the use of the energy produced by the PVG.

The integration of these two techniques, MPPT-Bat and DTC, offers significant benefits. These include increased solar energy production, improved power quality and fast response. As a result, these improvements lay the foundation for more sustainable agricultural irrigation practices. The implementation of MPPT-Bat and DTC techniques results in reduced agricultural energy costs, extended irrigation system life and improved pumped water flow rates. In addition, increased solar energy production helps to reduce dependence on fossil fuels. This has a positive impact on the environment. Indeed, these results suggest that these techniques can overcome the limitations generally associated with conventional MPPT approaches. As a limitation of this approach, it should be noted that, according to the results obtained, there is a fluctuation in the electromagnetic torque. These fluctuations can be reduced by using intelligent estimation techniques. Also, the speed can be estimated to avoid the use of the speed sensor, which would make the system even more advantageous. These limitations will be the subject of our future research.

## Supporting information

S1 Dataset (RAR)

## Abbreviations

*DTC*: Direct Torque Control
PSO: Particle Swarm Optimization
*FBAC*: Firefly Based Ant Colony
*SWPS*: Solar Water Pumping System
*INC*: Incremental Conductance
*P&O*: Perturb and Observe
*PV*: Photovoltaic
*PVG*: Photovoltaic Generator
*D*_*min*, *max*_: Minimum and Maximum Duty cycle
*V*_*oc*_: Open-Circuit Voltage
*I*_*sc*_: Short-Circuit Current
*MPP*: Maximum Power Point
*MPPT*: Maximum Power Point Tracking
FOCV: Fractional Open-Circuit Voltage
FoFL: Fractional-Order Fuzzy Logic
N: Particle Number
f: Frequency
*x*_*i*_: Position
*v*_*i*_: Velocity
*(v*_*α*,_, *v*_*β*_*)*: Components of voltage in (α,β) reference frame
*(i*_*α*_, *i*_*β*_*)*: Components of current in (α,β) reference frame
*(ψ*_*α*_, *ψ*_*β*_*)*: Components of flux in (α,β) reference frame
*v*_*a*_, *v*_*b*_,: Phase simple voltages
*H*_*1*,*2*,*3*_: Switching states
*R*_*r*_, *R*_*s*_: Rotor and stator resistances
*L*_*m*_, *L*_*s*_, *L*_*r*_: Mutual, stator and rotor inductances
*p*: Pole pair number
*σ*: Leakage coefficient
*ω*, *Ω*: Mechanical pulsation and rotation speed of the machine
*T*_*em*,_ *T*_*r*_: Electromagnetic and load torque
*V*_*dc*_: DC bus voltage
*f*, *J*: viscous friction and total inertia
*θ*_*s*_: Stator flux position
